# Isolated compounds from *Dracaena angustifolia* Roxb and acarbose synergistically/additively inhibit α-glucosidase and α-amylase: an in vitro study

**DOI:** 10.1186/s12906-022-03649-3

**Published:** 2022-07-02

**Authors:** Jiling Yi, Ting Zhao, Yuanlin Zhang, Yanxing Tan, Xiao Han, Yulin Tang, Guangying Chen

**Affiliations:** 1 Key Laboratory of Tropical Medicinal Resource Chemistry of Ministry of Education, College of Chemistry and Chemical Engineering, Hainan Normal University, Haikou, 571158 People’s Republic of China; 2 Key Laboratory of Tropical Medicinal Plant Chemistry of Hainan Province, College of Chemistry and Chemical Engineering, Hainan Normal University, Haikou, 571158 People’s Republic of China

**Keywords:** *Dracaena angustifolia* Roxb, Acarbose, α-Glucosidase, α-Amylase

## Abstract

**Background:**

As a traditional herbal medicine, *Dracaena angustifolia* Roxb has been used as an anti-inflammatory agent by the Li people in Hainan, China. In preliminary phytochemical studies conducted in our lab, its fractions were found to inhibit α-glucosidase in vitro, indicating a potential for alleviating glucose dysregulation.

**Methods:**

Through in vitro enzymatic assays, the abilities of the separated components to affect α-glucosidase and α-amylase were evaluated. By establishing concentration gradients and generating Lineweaver–Burk plots, the corresponding inhibition modes together with kinetic parameters were assessed. Following the evaluation of the outcomes of their combination with acarbose, computational docking and molecular dynamic simulations were carried out to analyse the interaction mechanisms and perform virtual screening against human enzymes.

**Results:**

Compared with acarbose, 7 compounds, including flavonoid derivatives, amides and aromatic derivatives, with higher α-glucosidase inhibitory efficiencies were confirmed. It was found that those competitive/mixed candidates and acarbose interacted synergistically or additively on α-glucosidase. Moreover, 3 of them were able to inhibit α-amylase in mixed mode, and additive effects were observed in combination with acarbose. Through in silico docking, it was found that the active site residues as well as adjacent residues were involved in α-glucosidase and α-amylase binding, which were mainly achieved through hydrogen bonding. Among those dual-function flavonoids, Compound 9 was predicted to be a considerable inhibitor of human enzymes, as the formation of ligand–enzyme complexes was mediated by the residues responsible for substrate recognition and catalysis, the stabilities of which were reiterated by molecular dynamics simulations.

**Conclusion:**

Despite their mild effects on α-amylase, considerable α-glucosidase inhibitory efficiencies and potential synergy with acarbose were exhibited by these natural candidates. Furthermore, a stable ligand, human α-glucosidase, was predicted by the performed simulations, which provided useful information for the application of *Dracaena angustifolia* Roxb in diabetes treatment.

**Supplementary Information:**

The online version contains supplementary material available at 10.1186/s12906-022-03649-3.

## Introduction

Type II diabetes mellitus (T2DM) is a chronic disease that causes long-term body damage and mental issues. T2DM is diagnosed by persistent hyperglycaemia, which is caused by the insufficient secretion of insulin or insulin resistance [1]. Although multiple environmental or hereditary risk factors, as well as their complex correlations, have been identified in recent decades, the pathophysiology of T2DM is not completely understood [2]. For instance, a higher prevalence in the Asian population will be noted after reviewing worldwide epidemiological data; even though they are individuals with lower body mass indices, the incidence within this population is nearly twice the national average rate in America [3]. Similar vulnerability was also observed in China; the occurrence of diabetes (cases of type I diabetes included) has explosively increased from 0.67% in 1980 to 11.2% in 2017, and there are an estimated 129.8 million adults suffering from this disease, and the number is forecasted to be above 150 million by 2040 [4]. Currently, no permanent cure exists, and treatment and management are heavily dependent on medicine [5, 6]. For those who already have life-threatening complications, insulin therapy will be initiated to compensate for their impaired β-cell function, while the majority of patients could receive satisfactory outcomes using oral drugs. For example, acarbose, one of the most popular first-line agents in China and other developing countries, ameliorates hyperglycaemia by inhibiting the activities of α-glucosidase and α-amylase, which are key hydrolases of carbohydrate metabolism [7]. However, poorly digested oligosaccharides and starch will be fermented by colonic bacteria, and dyspepsia, flatulence, and diarrhoea are frequently reported after administration. Rarely, to avoid the risk of hepatotoxicity, the dosage should be adjusted for certain patients [8, 9].

Isolated compounds from pharmaceutical plants are considered to be alternatives. As indicated in clinical trials and laboratory studies, equivalent efficiency and improved safety could be ensured by monotherapy with those antidiabetic compounds or in combination with reduced acarbose [10, 11]. Furthermore, traditional medicine is capable of providing a source of natural candidates; for example, extract from mulberry leaf ameliorated insulin resistance in rodent models [12], and a hypoglycaemic effect was displayed by the ingredient from *Rehmannia glutinosa*, an essential constituent of Xiao Ke Wan (a Chinese traditional medicine formula for diabetes) [13]. Activities of α-glucosidase inhibition together with β-cell protection were observed in the crude/phenolic fractions from resina draconis, which is an ethnic minority medicine harvested from *Dracaena* plants [14, 15].

As a no-resin-producing species of the *Dracaena* genus, *Dracaena angustifolia* Roxb is an evergreen shrub widely distributed in tropical areas, and it is utilized by the Li people in China’s Hainan province as an herbal remedy [16]. According to documented folk recipes, the decoction prepared from its roots could be used for relieving abdominal pain, which might be related to the steroidal saponins from *D. angustifolia* Roxb [17, 18]. Our lab demonstrated that the flavonoids present in stems contributed to the therapeutic effect as well [19], and being able to suppress α-glucosidase in a dose-dependent manner was one of the features of fractions collected from previous phytochemical research (Supplementary Fig. 1). To exploit the potential of *D. angustifolia* Roxb in antidiabetic drug development, the IC_50_ values of each separated substance against α-glucosidase were initially evaluated. Compared to the reference acarbose, a total of seven compounds (5, 6, 8, 9, 18, 22, 24) with better inhibition efficiencies, ranging from 1.99 μM to 0.65 mM, were confirmed. Then, the corresponding standard curve and Lineweaver–Burke plots were constructed to investigate the corresponding inhibition models, and it was found that Compound 18 with the lowest IC_50_ and Compound 5 were an uncompetitive inhibitor and a noncompetitive inhibitor, respectively. Competitive or mixed-type inhibition was observed for Compounds 6, 8, 9, 22, and 24, all of which were able to exhibit synergistic effects with acarbose in the subsequent combination assay. Moreover, those five compounds were subjected to α-amylase activities assays, and only the flavonoids (Compounds 6, 8, 9) showed weak inhibitory actives, with IC_50_ values two orders of magnitude higher than that of acarbose (10 μM). As revealed by kinetic and synergism analyses, the three compounds acting as mixed inhibitors were able to enhance the inhibition of acarbose against α-amylase. The docking technique was employed to assess the possible molecular mechanism. In addition to the active site residues and the adjacent residues, HIS112, GLU411, and ASP442 were thought to contribute to the interactions between those small ligands and α-glucosidase. For porcine α-amylase, GLN63 was important for the inhibitory activities of Compounds 6 and 9. Then, docking was repeated to assess the compounds that displayed inhibitory effects on α-glucosidase and α-amylase, in which homologous enzymes of humans were chosen. Compound 9 generated the best configuration, by which four sites for substrate binding (ASP404, ARG600, ASP616, and HIS674) in human α-glucosidase, part of the catalytic sites and one residue for calcium binding (HIS201, ASP197, and GLU233) in human α-amylase were affected. With the information created by the molecular dynamics simulation, the stabilization of the human α-glucosidase–Compound 9 complex was confirmed. This study provides useful information for further in vivo research and antidiabetic drug discovery regarding *D. angustifolia* Roxb.

## Materials and methods

### Materials

**Fig. 1:**
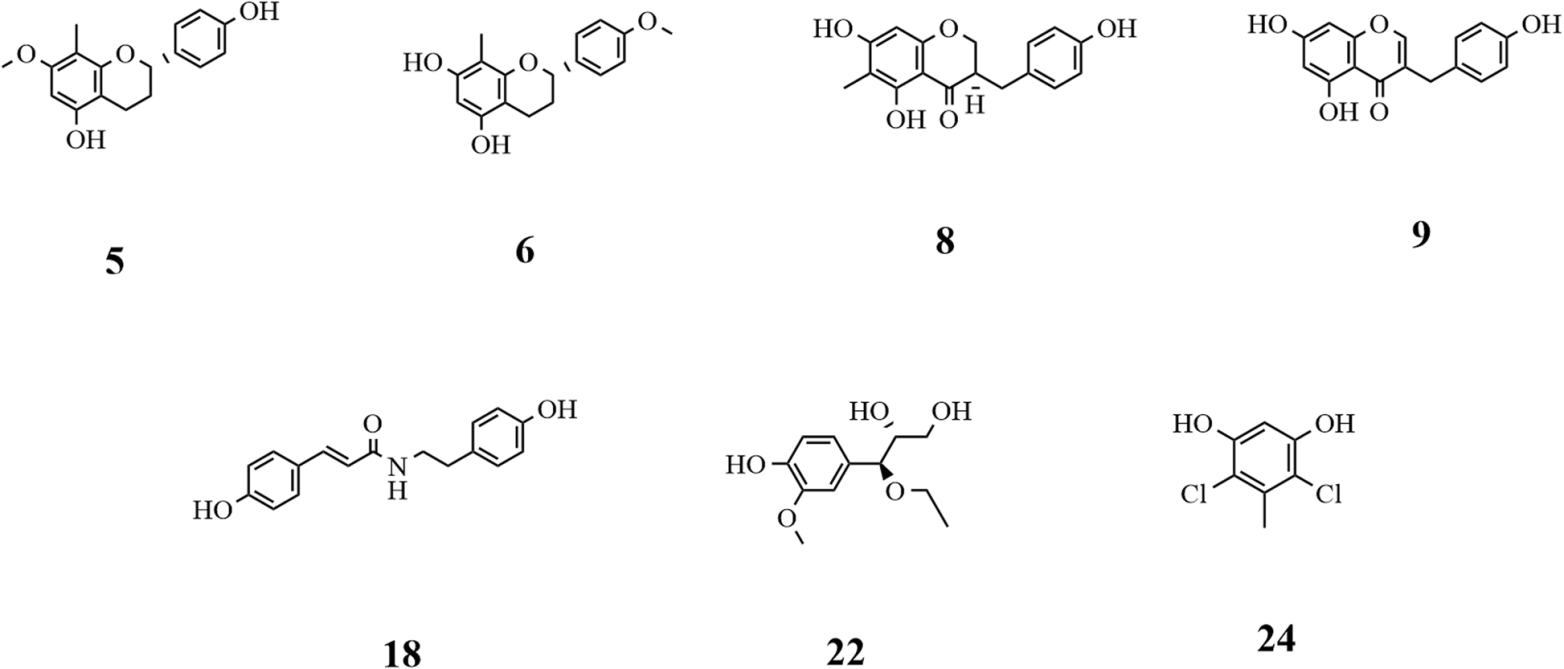
Structures of Compounds 5, 6, 8, 9, 18, 22, and 24, which were isolated from *D. angustifolia* Roxb

Bioactive compounds from the stems of *D. angustifolia* Roxb were prepared as reported by Zhao et al. [19]. (2S)-4′,5-Dihydroxy-7-methoxy-8-methylflavan (Compound 5), (2S)-4′-hydroxy-5,7-dimethoxy-8-methylflavan (Compound 6), 5,7-dihydroxy-6-methyl-3-(4′-hydroxybenzyl) chroman-4-one (Compound 8), 5,7-dihydroxy-3-(4′-hydroxybenzyl) chromone (Compound 9), trans-N-p-coumaroyltyramine (Compound 18), erythro-7R,8S-7-O-ethylguaiacyl glycerol (Compound 22), and 4,6-dichloro-5-methyl-benzene-1,3-diol (Compound 22) are listed in Fig. 1 (other structures are shown in Supplementary Fig. 2). Acarbose (A8980), α-glucosidase (*Saccharomyces cerevisiae*), and α-amylase (porcine pancreas) were purchased from Sigma–Aldrich. 4-Nitrophenol (pNP), 4-nitrophenol-α-D-glucopyranoside (pNPG), soluble starch from potato and other reagents were purchased from common commercial suppliers and used as received.

### α-Glucosidase inhibition assay

α-Glucosidase inhibitor screening was conducted according to a previously reported procedure with slight modifications [20]. A measured amount of pNPG powder was dissolved at 2 mg/mL in 0.01 M phosphate buffer (pH = 6.8), and then 50 μL of the pNPG solution was mixed with an equal volume of isolated compounds from *D. angustifolia* Roxb, which were diluted to 4 mM. After being kept at 37 °C for 10 min, 25 μL of α-glucosidase (0.5 U/mL) was added. Following another 10 min incubation at 37 °C, the reaction was stopped with 125 μL of 0.2 M Na_2_CO_3_, and the absorbance at 410 nm of each sample (converted pNP) was measured via a microplate reader (BioTek ELX-800, USA). A standard curve was generated using serially diluted pNP, acarbose and assay buffer as positive and blank controls. The percent inhibition was calculated by the following formula: % inhibition = (1 - A_Sample_ / A_Control_) × 100%. For the compound with a greater inhibition rate (vs. the same amount of acarbose), IC_50_ values were determined by plotting the concentration gradient against the corresponding inhibition percentage.

### α-Amylase inhibition assay

Changes in α-amylase activities were quantitatively analysed via a method based on starch-iodine colorimetric detection [21]. Soluble starch (0.4 g) was gently stirred in 80 mL PBS buffer (pH = 6.9), which was subsequently diluted to 100 mL as the substrate. A mixture of 40 μL of starch solution and 40 μL of selected compounds was maintained at 37 °C for 10 min, and 80 μL α-amylase (5 U/mL) was added to start the reaction. After 30 min at 37 °C, terminations were achieved using 40 μL of 1 M HCl. The presence of starch was visualized with 200 μL of iodine reagent (0.2% I_2_: 2% KI), and the optical density at 580 nm was directly proportional to its concentration. Their influences on α-amylase were calculated as percent inhibition according to the equation % inhibition = (A_Positive_ - A_Sample_)/(A_Positive_ - A_Blank_) × 100%, where A_Positive_ and A_Blank_ are the absorbance values of PBS buffer containing acarbose or without compounds from *D. angustifolia* Roxb. A starch calibration curve ranging from 0.0625 to 4 mg/mL and a plot of inhibition rate versus compound concentration were prepared to derive corresponding IC_50_ values.

### Kinetic tests for α-glucosidase and α-amylase

To acquire the inhibition type and related parameters of those inhibitors, kinetic assays were carried out, and the conditions of enzymatic reactions are detailed in the above section, in which the amount of enzymes was fixed. With an increasing amount of substrate, the amount of hydrolysed pNPG or starch in the presence of compounds (concentration gradient was established on the basis of their IC_50_ value) was quantified to create Lineweaver–Burk plots. The equations were rearranged into double reciprocal forms:

$$\frac{1}{v}=\frac{Km}{Vmax}\left(1+\frac{\left[I\right]}{Ki}\right)\times \frac{1}{\left[S\right]}+\frac{1}{Vmax\ }\ \left(\mathrm{Competitive}\ \mathrm{inhibition}\right)$$

$$\frac{1}{v}=\frac{Km}{Vmax}\times \left(1+\frac{\left[I\right]}{Ki}\right)\times \frac{1}{\left[S\right]}+\frac{1}{Vmax}\left(1+\frac{\left[I\right]}{Ki}\ \right)\ \left(\mathrm{Noncompetitive}\ \mathrm{inhibition}\right)$$

$$\frac{1}{v}=\frac{Km}{Vmax}\times \frac{1}{\left[S\right]}+\frac{1}{Vmax}\left(1+\frac{\left[I\right]}{Ki}\ \right)\ \left(\mathrm{Uncompetitive}\ \mathrm{inhibition}\right)$$

### Combined inhibition of acarbose and selected compounds

Three concentrations (nearly equivalent to 1/4 IC_50_, 1/2 IC_50_, IC_50_) of acarbose and chosen compounds were combined using freely available software (CompuSyn Version 1.0; https://www.combosyn.com/) [22]. A combination index (CI) was employed to evaluate the impacts of binary inhibitors on α-glucosidase and α-amylase. The fundamental equation was expressed as follows:

$$\mathrm{CI}=\frac{(D)1}{\left({D}_x\right)1}+\frac{(D)2}{\left({D}_x\right)2}$$

where (*D*)_1_ and (*D*)_2_ are the doses of sample and acarbose that inhibit a certain level of enzyme in the combination system, respectively, and (*D*_*x*_)_1_ and (*D*_*x*_)_2_ are the doses of a single inhibitor that produce the same level of inhibition. When the CI values were above 1.1, between 0.9 and 1.1, and below 0.9, the effects were defined as antagonistic, additive, and synergistic.

### In silico analyses

The mechanisms of action and possible interactions were predicted by AutoDock Vina [23]. Crystal structures of isomaltase (PDB ID: 3A4A), which shares 72% sequence similarity with the α-1,4-glucosidase, α-amylase (PDB ID: 1OSE), and their homologues in *Homo sapiens* (PDB IDs: 5NN8 and 2QV4) are available in the protein databank (www.rcsb.org/pdb). 3D structures with minimized energy of Compounds 6, 8, 9, 22, and 24 were sketched using Chem3D (PerkinElmer Informatics, CA). Prior to docking, water molecule removal, Gasteiger charge addition, and the addition of hydrogen were carried out. Compounds were incorporated as ligands, whose bond rotations were set as default. The docking region was placed in the sites covering the active residues. For the α-glucosidase from *Saccharomyces cerevisiae*, a grid box, with a spacing of 30 Å, was centred at the coordinates of 20.159, − 6.004, and 22.986. For porcine pancreatic α-amylase, the X, Y, and Z centres were 36.436, 37.487, and − 2.166, respectively, with a spacing of 30 Å. For enzymes in human saliva and the pancreas, docking boxes with a spacing of 30 Å were generated, whose centres were defined as − 14.312, − 38.186, and 95.627 and 12.805, 46.042, and 25.979, respectively. Subsequently, the Broyden-Fletcher-Goldfarb-Shanno algorithm was chosen, and 50 independent calculations were conducted. Enzyme-compound complexes with the lowest binding energies were extracted, and the superimposed diagrams were analysed using PyMOL 2.6 [24].

The human enzyme–Compound 9 complexes were subjected to molecular dynamics simulations. After being separated, human α-glucosidase and α-amylase were prepared using Gromacs [25], in which the AMBER99SB-ILDN force field was chosen. Compound 9 was submitted to the acpype online server for generating AMBER topological files [26]. Then, complexes were rebuilt and inserted in a box with a distance of 10 Å from the edge of the protein, which was solvated via TIP3P model water molecules. The system charge was neutralized by the addition of sodium ions and energetically minimized with the steepest descent minimization method. Under the isothermal-isochoric ensemble (NVT) and the isothermal-isobaric ensemble (NPT), the systems were heated to 310 K for 100 ps, and the total pressures were maintained at 1 bar for 100 ps. The final production had a total of 10 ns, whose total energy and the ligand root-mean-square deviation (RMSD) fitted to the protein backbone were analysed.

### Statistical analysis

Each experiment was performed in triplicate, and the data were interpreted as the mean ± standard deviation (SD). Statistical analysis was carried out by one-way analysis of variance (ANOVA) using Origin 9.0 software.

## Results and discussion

### α-Glucosidase activities and kinetic constants

**Fig. 2:**
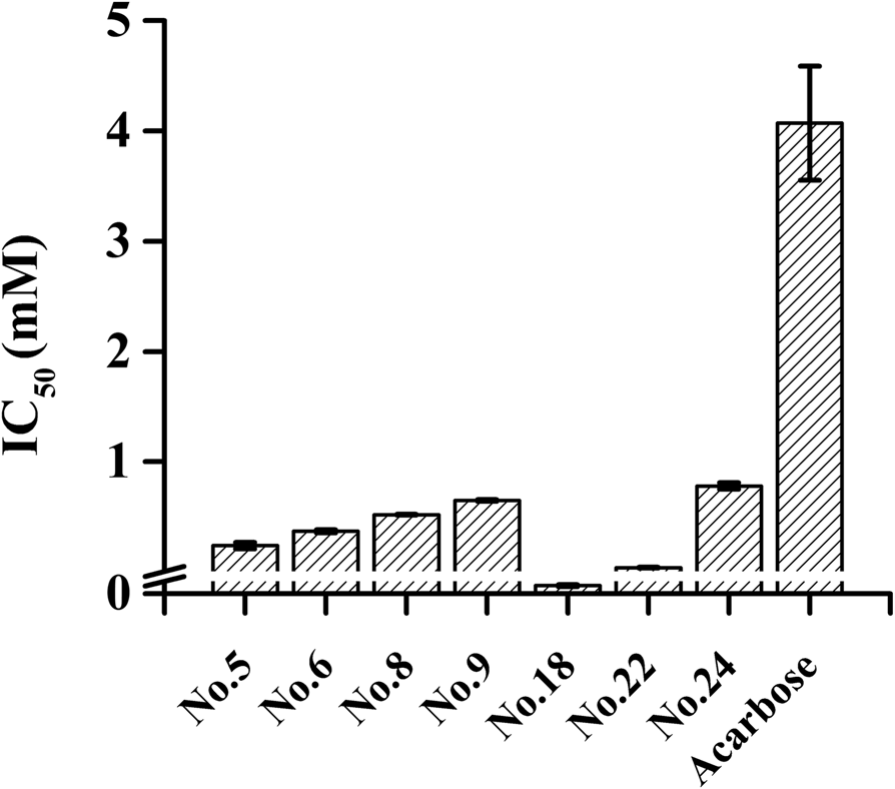
IC_50_ values of 7 selected compounds from *D. angustifolia* Roxb and acarbose against α-glucosidase in vitro

Despite the lack of scientific evidence confirming the genetic susceptibility to T2DM in the Asian population, a robust association between inappropriate dietary habits and the disease incidence is undoubtable [27]. Especially in China, the consumption of immoderate refined grain is quite common [28]. Therefore, discovering carbohydrate metabolism retardants or inhibitors from traditional medicine resources (herbs and plants) without apparent side effects is significant for T2DM prevention and treatment. As a continuation of our previous Li folk medicine modernization work, a total of 29 compounds derived from *D. angustifolia* Roxb were rendered for the preliminary assay, in which compounds with poor aqueous solubilities were excluded, and only 7 of them were found to have higher potency than acarbose (4.07 ± 0.516 mM). As shown in Fig. 2, their α-glucosidase inhibition capacities were characterized by their half maximal inhibitory concentrations. The IC_50_ values at low micromolar levels for Compounds 18 (1.99 ± 0.2 μM) and 22 (40 ± 4.0 μM), which are phenoic derivatives that have been briefly mentioned in the phytochemical screenings of *Crataegus pinnatifida* Bge and *Polygonum aubertii* Henry [29, 30], were illustrated, followed by Compounds 5, 6, 8, 9, and 24 with IC_50_ values of 0.24 ± 0.03 mM, 0.37 ± 0.02 mM, 0.52 ± 0.01 mM, 0.65 ± 0.01 mM, and 0.78 ± 0.03 mM, respectively.

**Fig. 3:**
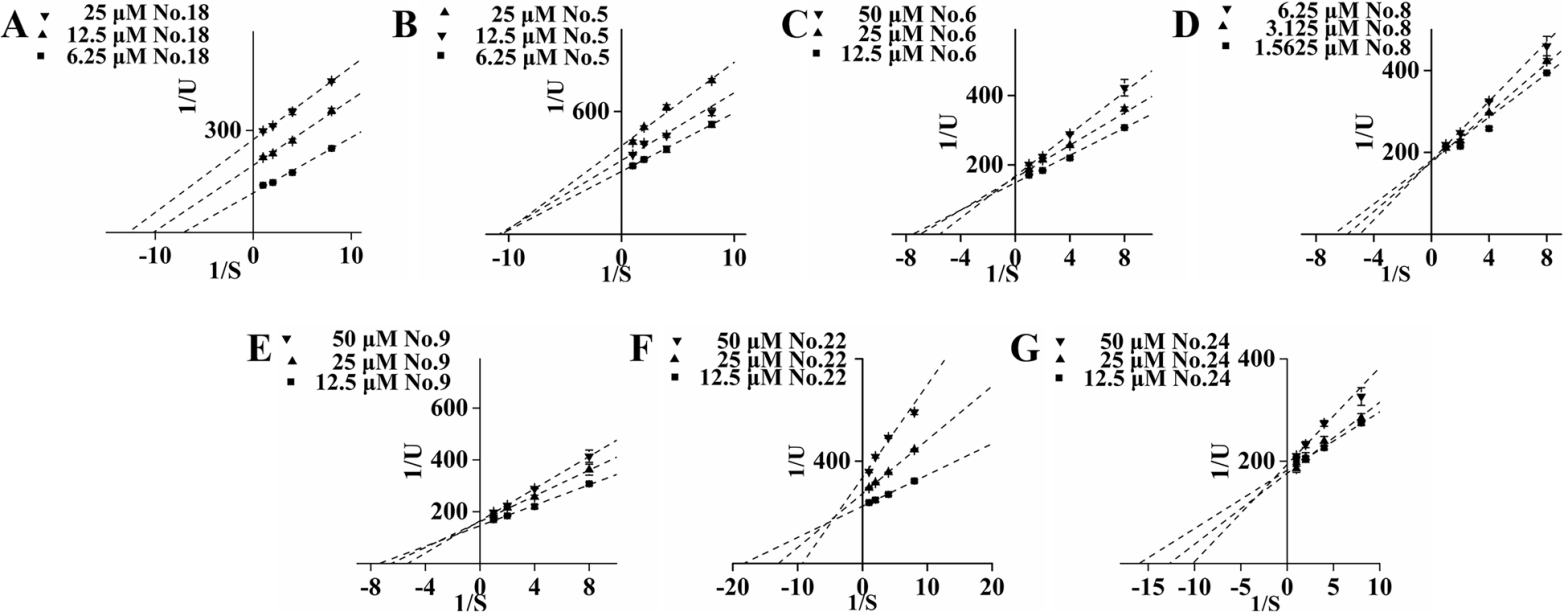
Lineweaver–Burk plots for kinetic analysis of α-glucosidase inhibition in the presence of Compounds 18 (**A**), 5 (**B**), 6 (**C**), 8 (**D**), 9 (**E**), 22 (**F**), and 24 (**G**)

Since the mechanism underlying the postprandial blood glucose control of acarbose and other approved α-glucosidase inhibitors is the targeting of the enzyme in a competitive manner, the corresponding reversibility should be emphasized. Modes of their inhibition were distinguished based on kinetic analyses, and it was demonstrated that the type for Compound 18 showing the most effective inhibitory activity was uncompetitive (Fig. 3A). As a rare class, it was very attractive with regard to novel drug design, while extra essential information, such as accurate or reliable binding patterns, was needed to investigate its potency. Compound 5, which was inferior, was defined as a noncompetitive inhibitor (Fig. 3B). Although some Indian and Indonesian medicine applied in T2DM treatment was attributed to the components acting as uncompetitive inhibitors, they were usually suitable for controlling the feedback regulation [31, 32].

In addition to the verification of the competitive behaviour of Compound 8, others were found to be mixed inhibitors. For a further comparison, their kinetic constants were calculated. As listed in Table 1, the smallest value of Ki in Compound 22 was noted, which reflected a greater affinity for α-glucosidase and more stable binding, despite a moderate IC_50_ value.

**Table 1:** Inhibition models and related parameters of selected glucosidase inhibitors from *D. angustifolia* Roxb

Compound	Mode of inhibition	Ki (μM)	Km (μM)
18	Uncompetitive	21.08 ± 12.25	0.12 ± 0.03
5	Noncompetitive	39.88 ± 8.47	0.14 ± 0.01
8	Competitive	11.16 ± 0.99	0.18 ± 0.01
6	Mixed	57.76 ± 1.67	0.17 ± 0.01
9	Mixed	54.42 ± 15.76	0.17 ± 0.01
22	Mixed	7.00 ± 1.97	0.04 ± 0.01
24	Mixed	43.96 ± 0.88	0.08 ± 0.01

### Combined effects of selected compounds and acarbose against α-glucosidase

**Fig. 4:**
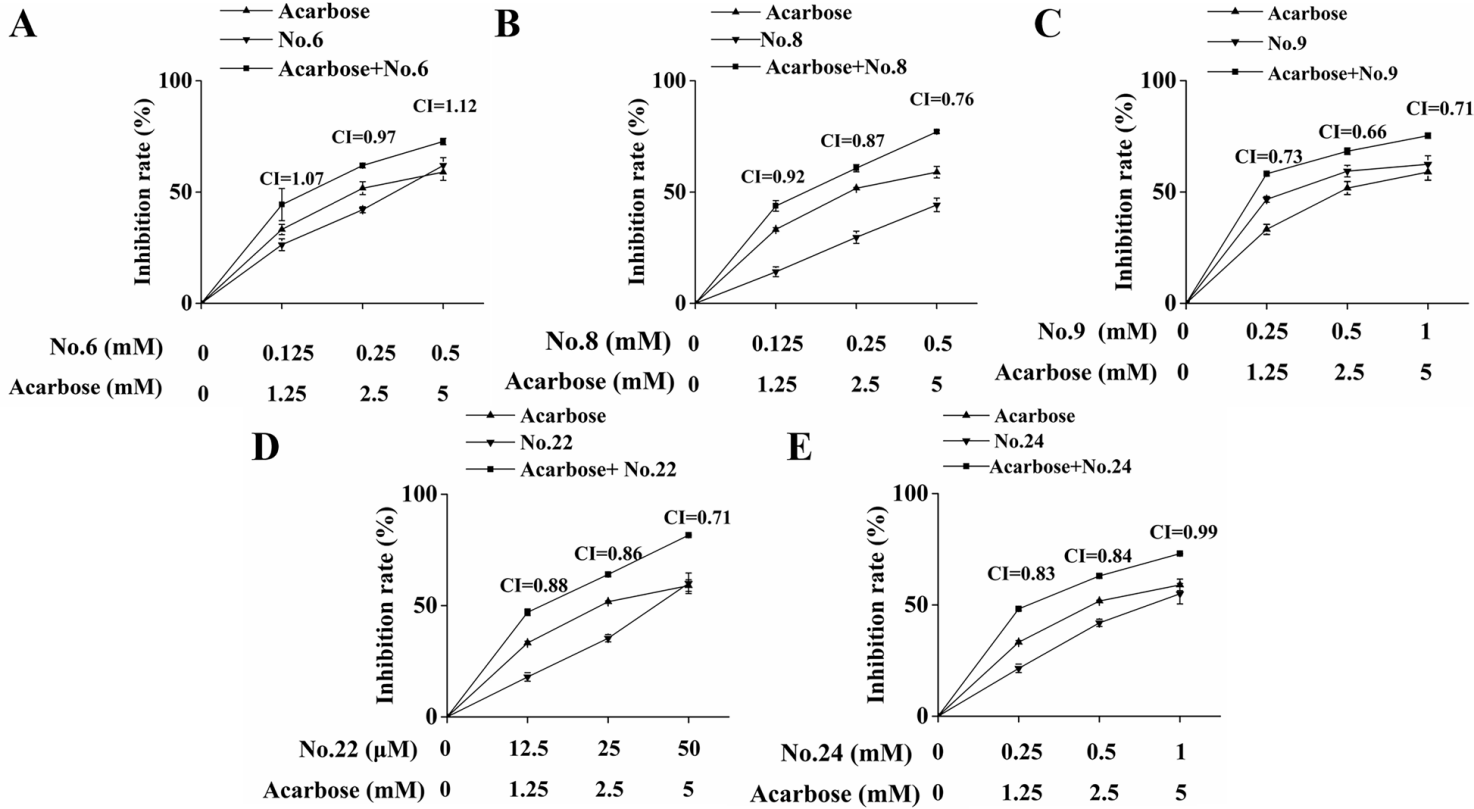
Inhibition rates of the combination of acarbose and Compounds 6 (**A**), 8 (**B**), 9 (**C**), 22 (**D**), and 24 (**E**) against α-glucosidase in vitro at gradient concentrations. The CI values above the data points were calculated by CompuSyn software

To identify candidates that could be potential substitutes for acarbose or complementary, the impacts of the combination of acarbose with competitive or partly competitive inhibitors from *D. angustifolia* Roxb on α-glucosidase activity were determined. As shown in Fig. 4, improved performance could be observed after the addition of those natural compounds. Following the concept of CI, the degree of interaction was investigated. For Compounds 8, 9, and 22, synergism could be concluded from those calculated values below 0.9, while moderate additive effects could be inferred from the increased CI values of the combination of Compound 24 and acarbose at the highest concentration. In the interaction between acarbose and Compound 6, a value (1.12) indicating slight antagonism was achieved.

### α-Amylase activities and kinetic constants

**Fig. 5:**
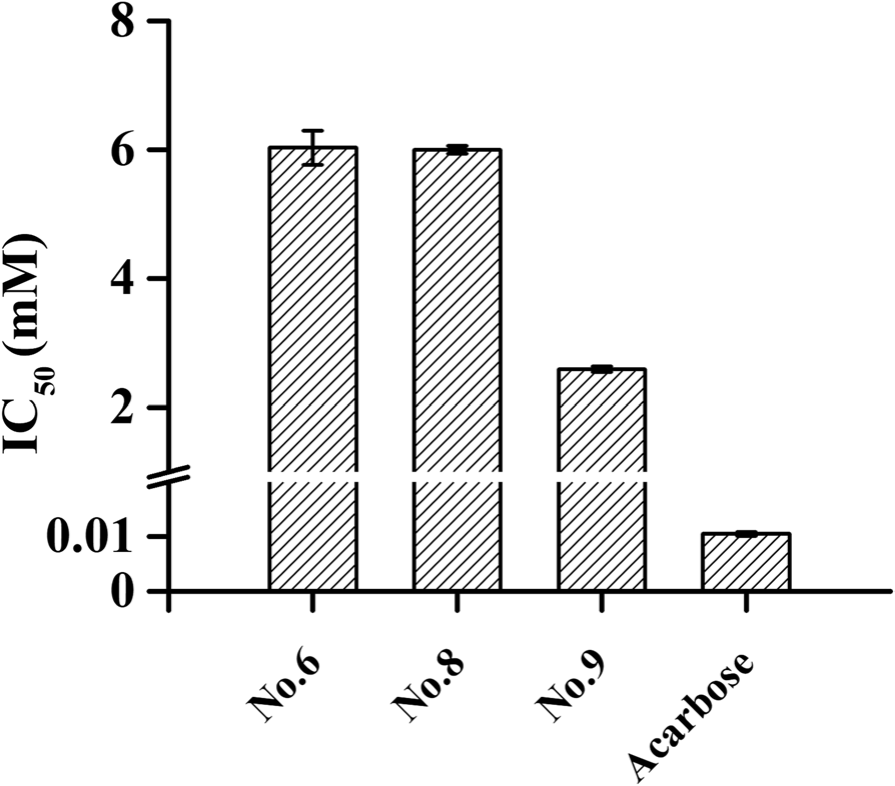
IC_50_ values of selected flavonoid compounds and acarbose against α-amylase in vitro

Considering the benefit of the prolonged digestion of polysaccharides for diabetes management, the influences of the tested natural α-glucosidase inhibitors on α-amylase activities in vitro were assessed, in which iodine solution was utilized for staining and quantifying the remaining starch. As mentioned in previous research, the 3,5-dinitrosalicylic acid (DNS) method usually used for the detection of converted maltose was found to be not acceptable [33]. The interference of the reducing potential of our phenolic compounds was also confirmed in the preliminary assay (data not shown). The IC_50_ value of acarbose against α-amylase was found to be 10.25 ± 0.01 μM, while mild α-amylase inhibition was recorded; only flavonoid compounds from *D. angustifolia* Roxb (6, 8, and 9) had IC_50_ values at the millimolar level (6.03 ± 0.27 mM, 5.99 ± 0.06 mM, and 2.59 ± 0.05 mM) (Fig. 5). In the test concentration range, Compounds 22 and 24 did not yield 50% inhibition.

**Fig. 6:**
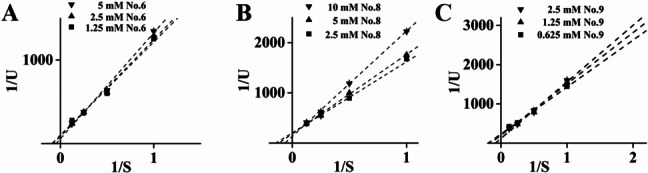
Lineweaver–Burk plots for the kinetic analysis of α-amylase inhibition in the presence of Compounds 6 (A), 8 (B), and 9 (C)

Then, serial dilutions from appropriate concentrations were performed to acquire related kinetic characteristics and parameters. They displayed competitive/noncompetitive mixed inhibitions on α-amylase, including Compound 8, the potential competitive α-glucosidase inhibitor (Fig. 6). Among them, Compound 9, with the lowest Ki and IC_50_, was supposed to be more probable (Table 2).

**Table 2:** Inhibition models and related parameters of selected flavonoids

Compound	Mode of inhibition	Ki (mM)	Km (mM)
6	Mixed	27.47 ± 3.51	14.01 ± 0.53
8	Mixed	14.12 ± 3.53	7.98 ± 0.46
9	Mixed	8.59 ± 1.94	6.39 ± 0.18

### Combined effects of selected flavonoids and acarbose against α-amylase

**Fig. 7:**
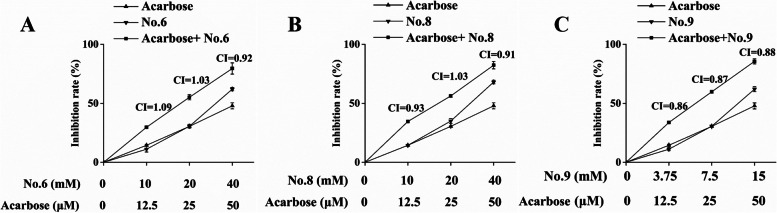
Inhibitory effects of the combination of acarbose and Compounds 6 (**A**), 8 (**B**), and 9 (**C**) against α-amylase. The CI values above the data points were calculated by CompuSyn software

Despite low efficiency, the in vitro α-amylase inhibition assay was repeated, in which each of the selected flavonoids were mixed with acarbose, and the corresponding conclusion was determined. Similar to the results obtained from α-glucosidase inhibitors, lower α-amylase activities were observed (Fig. 7). The generated CI values, which were in the range of 0.9 and 1.1, indicated that higher inhibitory rates were attributed to the additive effects of Compounds 6, 8, and 9.

### Molecular docking and dynamics simulations

**Fig. 8:**
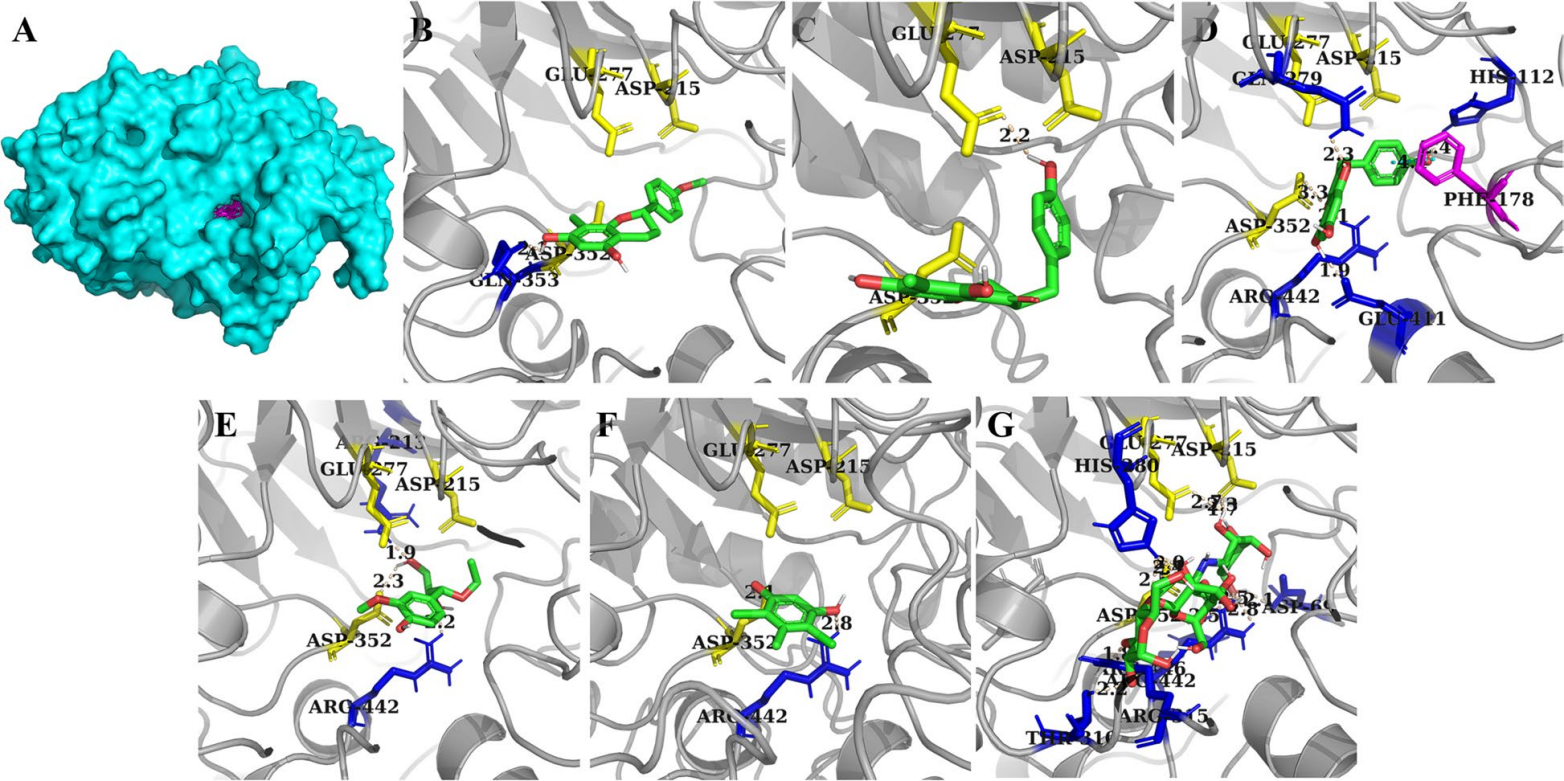
α-Glucosidase docking results of all compounds (**A**), Compounds 6 (**B**), 8 (**C**), 9 (**D**), 22 (**E**), 24 (**F**), and acarbose (**G**), in which catalytic sites, residues involved in the formation of hydrogen bonds and pi-pi interactions are indicated in yellow, blue, and magenta

Due to the competitive or competitive/noncompetitive inhibition modes, the natural inhibitors separated from *D. angustifolia* Roxb would interact with the catalytic core of α-glucosidase or α-amylase during the carbohydrate-hydrolase reactions. To gain insights into the binding properties of these bioactive substances to the enzyme, the involved sites, residues, hydrogen bonds, and pi-pi interactions were analysed via computational docking technology. After being prepared as ligands, 50 successful docking runs were processed, and the conformation with minimal binding energy was provided as the reliable prediction. As shown in Fig. 8, compounds were buried in the pocket of α-glucosidase, and pi-pi interactions were only predicted between the side chains of PHE178 and Compound 9.

More details about the docking results are listed in Supplementary Table 1. It was suggested that the weakest affinity was obtained by Compound 24, in which ARG442 and active residue ASP352 were involved. A similar binding pattern was detected for Compounds 22 and 9, and the smallest Ki of the former compound might be contributed by shorter hydrogen bonds. For Compound 9, the lowest binding energy was displayed, as the interactions with three more residues (HIS112, GlN279, and GLU411) were revealed by docking. Among those two compounds that have better scores than acarbose, only one reliable hydrogen bond was visualized by the analysis tool, catalytic resides GLU277 for Compound 8 and GLN353, which was adjacent to key residue ASP52, for Compound 6.

**Fig. 9:**
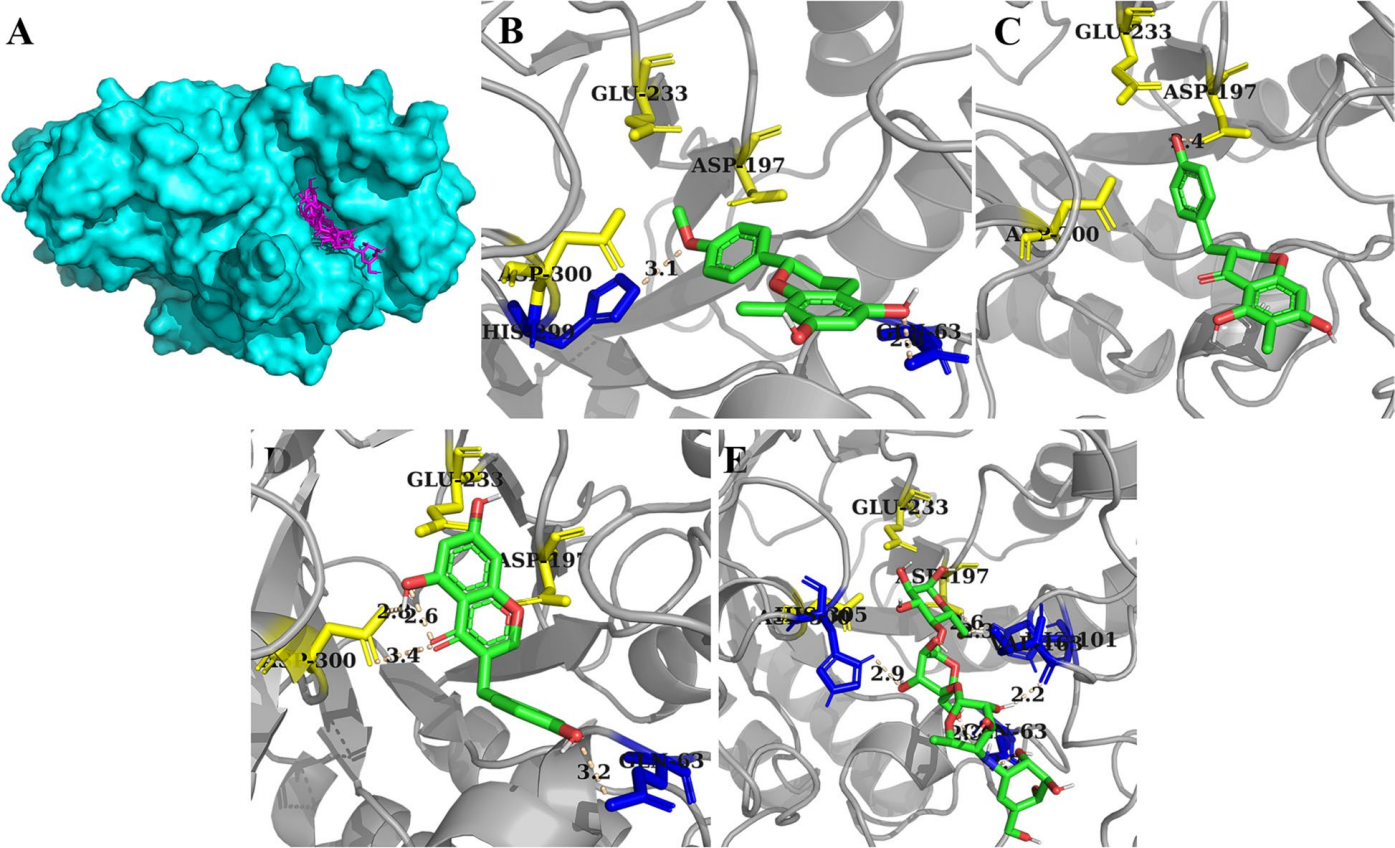
α-Amylase docking results of all compounds (**A**), Compounds 6 (**B**), 8 (**C**), 9 (**D**), and acarbose (**D**), in which catalytic sites and residues involved in the formation of hydrogen bonds are illustrated in yellow and blue

For the case of α-amylase binding, pi-pi stacking between the aromatic side chains of TRP59, Tyr62, and those flavonoid derivatives was illustrated (Fig. 9), by which the position of Compound 8 was partly restricted, and only catalytic ASP197 was found in its docking results. With an increasing degree of unsaturation, Compound 9, with a more rigid structure, obtained a stable binding configuration with ASP300. The weak inhibitory effect of Compound 6 on porcine α-amylase might be attributed to the lack of direct interaction with catalytic residues, in which GLN63 and HIS299 were involved (Supplementary Table 2).

**Fig. 10:**
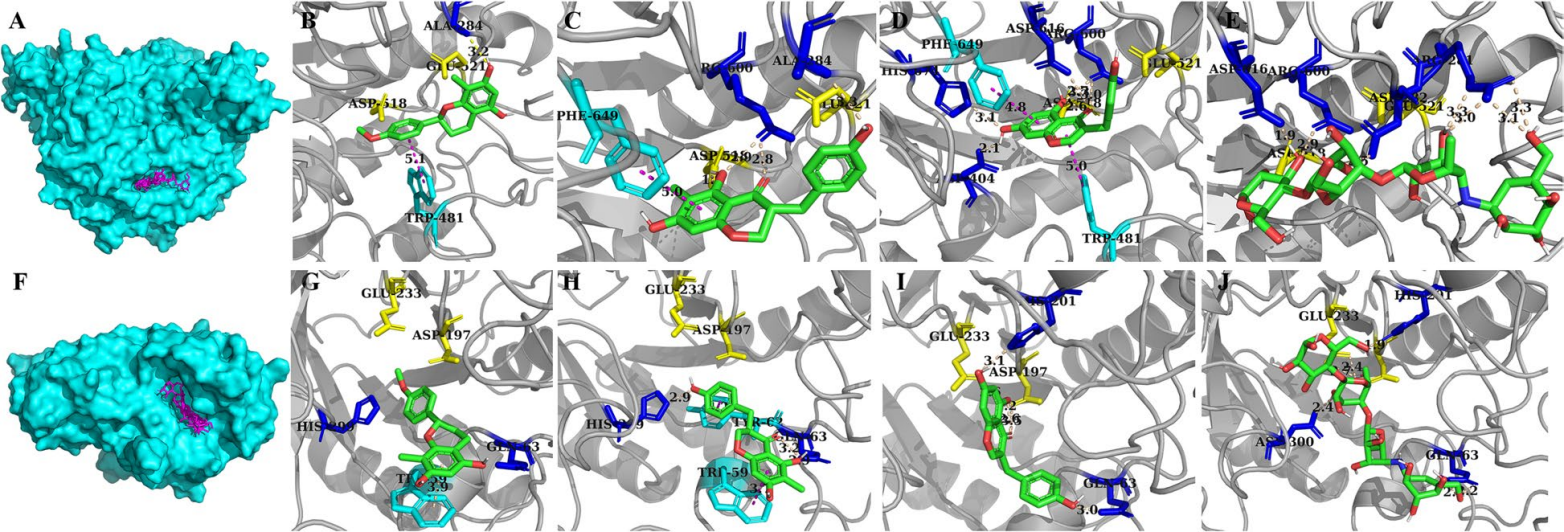
Human α-glucosidase docking results of all compounds (**A**), Compounds 6 (**B**), 8 (**C**), 9 (**D**), and acarbose (**E**); human α-amylase docking results of all compounds (**F**), Compounds 6 (**G**), 8 (**H**), 9 (**I**), and acarbose (**J**). The catalytic sites, residues involved in the formation of hydrogen bonds and pi-pi interactions are indicated in yellow, blue, and magenta

Considering that enzymes participating in poly/oligo-saccharide metabolism in the human body are the real targets that need to be affected, the structural differences between them and the protein utilized in the experiment were the key factors determining the reliability of the results generated by the above assays. Those compounds capable of repressing nonhuman α-glucosidase and α-amylase simultaneously were redocked, in which acarbose was included as the reference. The presumed docking configurations are shown in Fig. 10. The pi-pi interactions between TRP481 and the PHE649 of α-glucosidase and those ligands was demonstrated. For α-amylase, the side chain of TRP59 was essential for the formation of pi-pi stacking, the strength of which was moderate.

Moreover, docking scores together with the patterns of hydrogen bonds of those flavonoids indicated that Compound 9 was a considerable antidiabetic candidate; it might occupy all of the substrate binding sites (ASP404, ARG600, ASP616, and HIS674) of α-glucosidase and all of the active site residues (ASP197 and GLU233) of α-amylase (Supplementary Table 3).

**Fig. 11:**
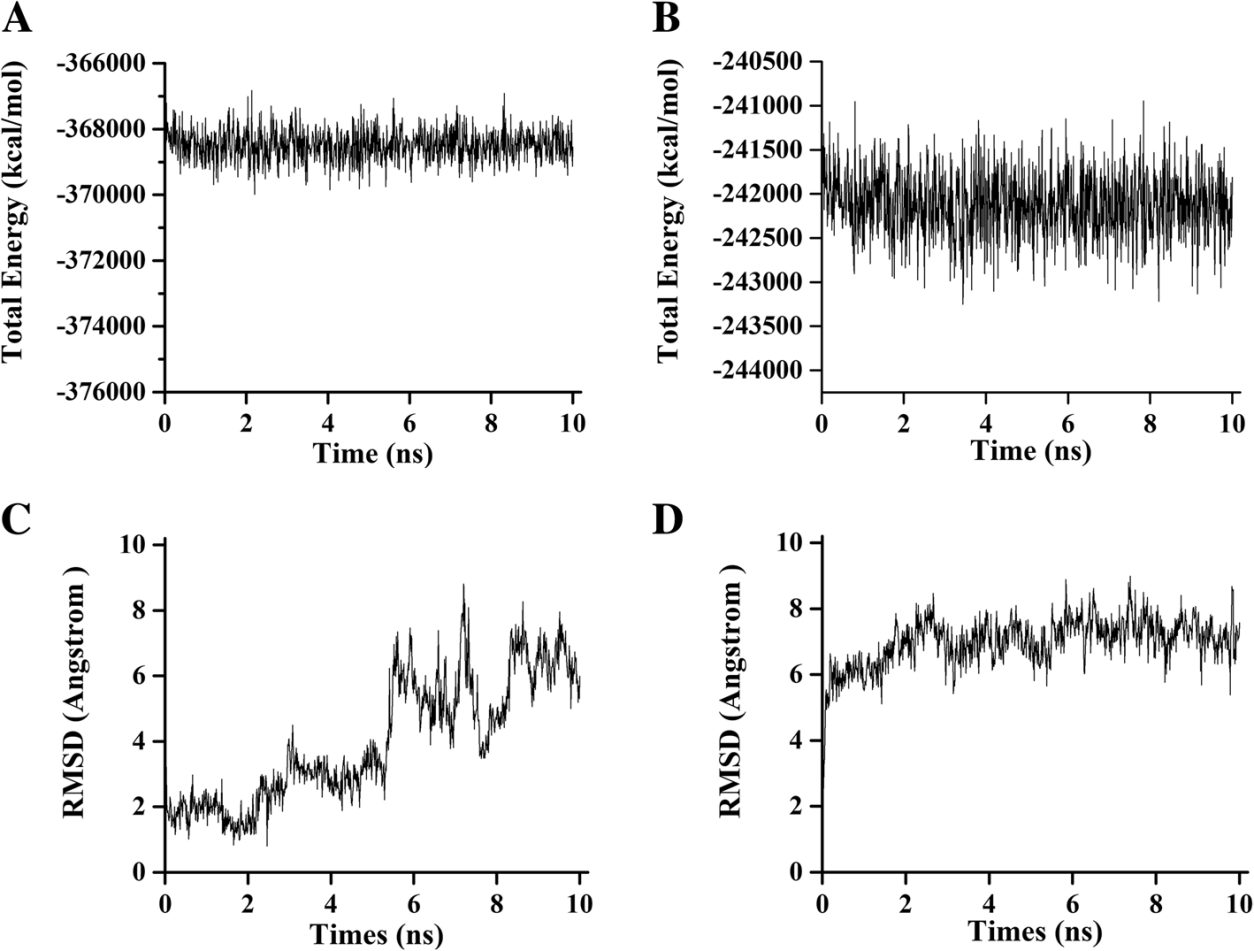
Molecular dynamics simulation results of human α-glucosidase compound-9 (**A** & **C**) and human α-amylase-Compound 9 (**B** & **D**)

After further analysis by molecular dynamics simulations, the average energy of the total system was − 368,484.7 Kcal/mol (α-glucosidase–Compound 9) and − 242,119.5 Kcal/mol (α-amylase–Compound 9). The details of the corresponding energy graphs, having minimal variations, are displayed in Fig. 11A&B. According to the corresponding RMSD values, a change in the binding mode of Compound 9 for α-glucosidase was observed (0.11 nm to 0.69 nm), which was located in the catalytic pocket and linked with ASP404 (Supplementary Fig. 3). Then, there was a general uniform stability. Moreover, the original position of Compound 9 in α-amylase was maintained until 5 ns, and the sharp increase in RMSD indicated that part of Compound 9 left significant residues, which was initiated by the formation of hydrogen bonds with the solvent (Fig. 11C&D).

## Conclusions

As a representative of Li folk medicine, the bacteriostatic, antitumor, and anti-inflammatory features of *D. angustifolia* Roxb have been verified, in which the functions of bioactive components are strongly related to their chemical structures. The previous phytochemical assay and the preliminary assessment of enzyme inhibition recalled the existence of substances that might interrupt carbohydrate digestion. In subsequent research, more efficient α-glucosidase inhibitors (Compounds 5, 6, 8, 9, 22, and 24) were identified (with acarbose as a reference) and were proven to exhibit uncompetitive, competitive, noncompetitive, or mixed manners. While their abilities to inhibit α-amylase activities were unremarkable, only three of them (Compounds 6, 8, and 9) were considered to be bifunctional. As revealed in the computational docking, the mechanism of α-glucosidase inhibition was attributed to binding to residues HIS112, ARG213, GLU277, GLN279, ASP352, GLU411, and ARG442. Relatively weak hydrogen bonds with residues GLN63, ASP197, HIS299, and ASP300 were essential for α-amylase inhibition. Among compounds with dual inhibitory activities, Compound 9 was characterized by good redocking performance. Despite detecting a higher variation in RMSD values of human α-amylase–Compound 9, the possible human α-glucosidase inhibition of this compound isolated from *D. angustifolia* Roxb is worth being investigated in future research.

## Supplementary Information

**Additional file 1.**

## Data Availability

The datasets generated and/or analyzed during the current study are available from the corresponding author on reasonable request.
