# Supplementary material for: Isolated compounds from Dracaena angustifolia Roxb and acarbose synergistically/additively inhibit α-glucosidase and α-amylase: an in vitro study
Source: BMC Complement Med Ther. 2022 Jul 2;22:177. doi: 10.1186/s12906-022-03649-3 (PMC9250238; doi:10.1186/s12906-022-03649-3)
Supplement: Supplementary file 1 — Additional file 1. [file 12906_2022_3649_MOESM1_ESM.docx]

Supplementary Figure 1. Inhibitory activities of acarbose, crude extract of *Dracaena angustifolia* Roxb, extractions prepared via petroleum ether, ethyl ethanoate, and water on the α-glucosidase, whose final concentrations were adjusted to 2 mg/mL





Supplementary Figure 2. Structures of separated compounds from *Dracaena angustifolia* Roxb





Supplementary Figure 3. pNP concentration standard curve for the in vitro α-glucosidase assay.





Supplementary Figure 4. Soluble starch concentration standard curve for in vitro α-amylase assay.


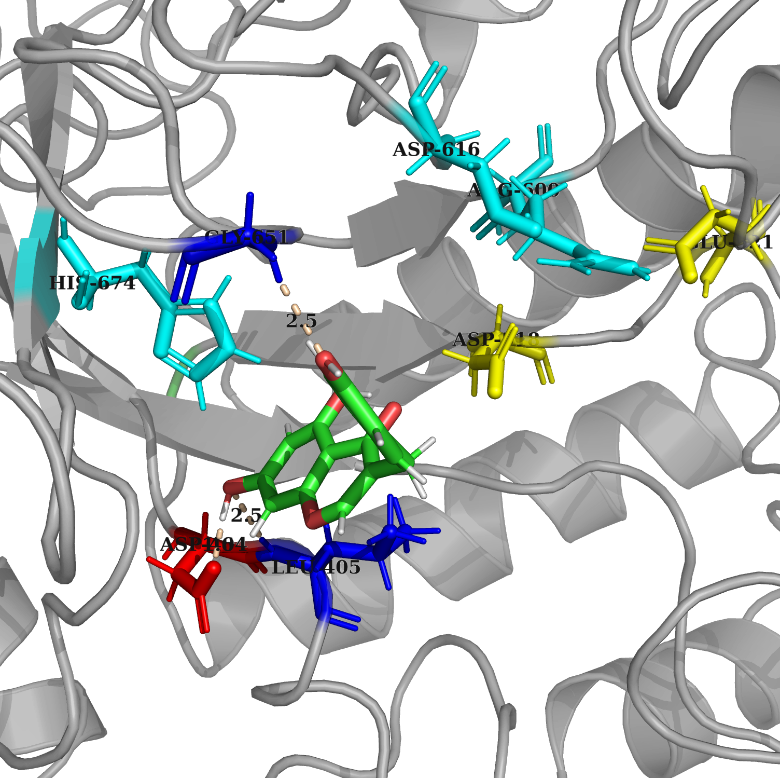


Supplementary Figure 5. The structure of human α-glucosidase-Compound 9 after 5 ns simulation, ASP404, which is responsible for substrate binding, was indicated as red. Other residues of this category as well as the active sites were illustrated by yellow and cyan.

Supplementary Table 1 The hydrogen bonds of compounds and acarbose in the α-glucosidase dockings

| **Compounds** | **Affinity (kcal/mol)** | **Protein-ligand interactions** | | | | |
| --- | --- | --- | --- | --- | --- | --- |
|  |  | **Ligand** | **Receptor** | **Interactions** | **Distances (Å)** | |
| No.6 | -8.1 | H36 | OE1-GLN353 | HBD | | 2.1 |
| No.8 | -7.7 | H38 | OE1-GLU277* | HBD | | 2.2 |
| No.9 | -8.2 | H31 | OE2-GLU411 | HBD | | 1.9 |
|  |  | O2 | 1HH1-ARG442 | HBA | | 2.1 |
|  |  | O2 | OD2-ASP352* | HBA | | 3.3 |
|  |  | O1 | 1HE2-GLN279 | HBA | | 2.3 |
|  |  | O5 | HE2-HIS112 | HBA | | 2.4 |
| No.22 | -6.3 | O3 | 2HH1-ARG442 | HBA | | 2.2 |
|  |  | H27 | OD2-ASP352* | HBD | | 2.3 |
|  |  | O2 | 1HH2-ARG213 | HBA | | 1.9 |
| No.24 | -5.8 | O3 | 2HH1-ARG442 | HBA | | 2.8 |
|  |  | H17 | OD2-ASP352* | HBD | | 2.1 |
| Acarbose | -6.8 | O15 | HG1-THR310 | HBA | | 2.2 |
|  |  | O12 | 2HH1-ARG315 | HBA | | 1.8 |
|  |  | O17 | HE2-HIS280 | HBA | | 2.4 |
|  |  | O4 | HE2-HIS280 | HBA | | 2.5 |
|  |  | O9 | HE2-HIS280 | HBA | | 2 |
|  |  | O5 | 1HH1-ARG442 | HBA | | 2.5 |
|  |  | O5 | 2HH1-ARG442 | HBA | | 2.8 |
|  |  | O5 | 1HH2-ARG446 | HBA | | 2.2 |
|  |  | H77 | OE1-GLU277* | HBD | | 2.7 |
|  |  | H77 | OD1-ASP215* | HBD | | 3.3 |
|  |  | H82 | OD1-ASP215* | HBD | | 1.7 |
|  |  | H87 | OD1-ASP69 | HBD | | 2.1 |

* All of three catalytic residues of *S. cerevisiae* α-glucosidase

Supplementary Table 2 The hydrogen bonds of compounds and acarbose in the α-amylase dockings

| **Compounds** | **Affinity (kcal/mol)** | **Protein-ligand interactions** | | | |
| --- | --- | --- | --- | --- | --- |
|  |  | **Ligand** | **Receptor** | **Interactions** | **Distances (Å)** |
| No.6 | -8.9 | O2 | NE2-GLN63 | HBA | 2.8 |
|  |  | O4 | NE2-HIS299 | HBA | 3.1 |
| No.8 | -9 | H38 | OD1-ASP197 | HBD | 2.4 |
| No.9 | -8.4 | H31 | OD2-ASP300 | HBD | 2.8 |
|  |  | O2 | OD1-ASP300 | HBA | 3.4 |
|  |  | O5 | NE3-GLN63 | HBA | 3.2 |
| Acarbose | -7.3 | H86 | OD2-ASP197 | HBD | 2.6 |
|  |  | O17 | HE2-HIS101 | HBA | 2.3 |
|  |  | O7 | HD1-HIS`305 | HBA | 2.9 |
|  |  | O14 | 1HE2-GLN63 | HBA | 2.3 |
|  |  | O10 | 2HE2-GLN63 | HBA | 2.7 |
|  |  | H72 | O-VAL163 | HBD | 2.2 |

* Two of three catalytic residues of porcine α-amylase

Supplementary Table 3 The hydrogen bonds of compounds and acarbose in the human α-glucosidase docking and human α-amylase dockings

| Human alpha-glucosidase | | | | | |
| --- | --- | --- | --- | --- | --- |
| Compounds | Affinity (kcal/mol) | Protein-ligand interactions | | | |
|  |  | Ligand | Receptor | Interactions | Distances (Å) |
| No.6 | -7 | O3 | N-ALA284 | HBA | 3.2 |
| No.8 | -7.2 | O3 | NH1-ARG600 | HBA | 2.9 |
|  |  | O2 | NH1-ARG600 | HBA | 2.8 |
|  |  | O5 | N-ALA284 | HBA | 3.3 |
| No.9 | -7 | O4 | NE2-HIS674 | HBA | 3.1 |
|  |  | H32 | OD2-ASP404 | HBD | 2.1 |
|  |  | O3 | NH1-ARG600 | HBA | 2.7 |
|  |  | O2 | NH1-ARG600 | HBA | 3 |
|  |  | O2 | OD2-ASP616 | HBA | 3.3 |
| Acarbose^a^ | -6.9 | O12 | NH1-ARG600 | HBA | 2.9 |
|  |  | H81 | OD2-ASP616 | HBD | 1.9 |
|  |  | H74 | OD2-ASP282 | HBD | 2.5 |
|  |  | O6 | NH2-ARG281 | HBA | 3 |
|  |  | O18 | NH2-ARG281 | HBA | 3.1 |
|  |  | O6 | NE-ARG`281 | HBA | 3.3 |
|  |  | O18 | NH1-ARG281 | HBA | 3.3 |
| Human alpha-amylase | | | | | |
| Compounds | Affinity (kcal/mol) | Protein-ligand interactions | | | |
|  |  | Ligand | Receptor | Interations | Distances (Å) |
| No.6 | -8.5 | O2 | NE2-GLN63 | HBA | 3.2 |
|  |  | O4 | NE2-HIS299 | HBA | 3.5 |
| No.8 | -8.9 | O3 | NE2-GLN63 | HBA | 2.9 |
|  |  | O2 | NE2-GLN63 | HBA | 3.2 |
|  |  | O5 | NE2-HIS299 | HBA | 2.9 |
| No.9 | -8.2 | O4 | NE2-HIS201 | HBA | 3.1 |
|  |  | O3 | OE1-GLU233* | HBA | 2.6 |
|  |  | O3 | OD1-ASP197* | HBA | 2.2 |
|  |  | O2 | OD1-ASP197* | HBA | 3.5 |
|  |  | O5 | NE2-GLN63 | HBA | 3 |
| Acarbose | -7.4 | H83 | OE1-GLU233* | HBD | 2.4 |
|  |  | H83 | OE2-GLU233* | HBD | 2.7 |
|  |  | H73 | OD2-ASP300 | HBD | 2.4 |
|  |  | O17 | HE2-HIS201 | HBA | 1.9 |
|  |  | H87 | OE1-GLN63 | HBD | 2.2 |
|  |  | O18 | 1HE2-GLN63 | HBA | 2.4 |

^a^ In the human alpha-glucosidase docking results, the effect on the active site residues was not detected by Pymol2. Reviewing the original position of acarbose in the crystal structure (5NN8), it was found such kind of interactions were mediated by water molecules.

* All of three catalytic residues of human α-amylase
